# Molecular Profiling of a Hepatocellular Neoplasm Not Otherwise Specified (HCN-NOS) Demonstrates Distinct Molecular Features in Hepatoblastoma and HCC-Like Components

**DOI:** 10.1177/10935266231204788

**Published:** 2023-10-30

**Authors:** Yan Chen Wongworawat, Stephen F. Sarabia, Martin Urbicain, Paola Francalanci, Pavel Sumazin, Rita Alaggio, Dolores H. López-Terrada

**Affiliations:** 1 Department of Pathology & Immunology, Baylor College of Medicine, Houston, TX, USA; 2 Department of Pathology, Texas Children’s Hospital, Houston, TX, USA; 3 Department of Pathology, Ospedale Pediatrico Bambino Gesù, Rome, Italy; 4 Department of Pediatrics, Baylor College of Medicine, Houston, TX, USA

**Keywords:** hepatoblastoma (HB), hepatocellular carcinoma (HCC), hepatocellular neoplasm-not otherwise specified (HCN-NOS), hepatoblastoma with carcinoma features (HBCs), immunohistochemistry (IHC)

## Abstract

Hepatoblastomas (HB) are embryonal tumors with quiet genomes diagnosed mostly in children under 3 years of age and often cured by surgical resection and chemotherapy. However, a subset of HBs behave aggressively, displaying characteristic histologic features and higher genomic instability. Hepatocellular neoplasm-not otherwise specified (HCN-NOS) is a provisional diagnostic category for tumors exhibiting either intermediate or a combination of both HB and hepatocellular carcinoma (HCC) histological features. In this study, we characterized an HCN-NOS diagnosed in a 3-year-old patient presenting with a liver mass, in which both HB and HCC histological components were amendable to macro-dissection and molecular profiling. The spectrum of mutations, copy number changes, mRNA, and protein expression profiles within these 2 histologically distinct tumor areas demonstrate molecular heterogeneity and suggest intratumoral clonal evolution of this hepatocellular *CTNNB*1-mutant lesion.

## Introduction

Hepatocellular malignant neoplasm not otherwise specified (HCN-NOS) is a provisional entity included in the Pediatric Liver Tumors International Consensus classification and recently in the WHO (World Health Organization), created to designate difficult to classify pediatric hepatocellular tumors with histological features that are either intermediate or contain distinct areas displaying HB and HCC histological features.^1,2^ HCN-NOS are aggressive tumors often diagnosed in older children with characteristic histological and, recently reported molecular features.^3,4^ The utility of immunohistochemical (IHC) stains to support the diagnosis is an area of active investigation.^3-5^ In this case report, we illustrate the distinct histopathology, IHC, cytogenomic, and mRNA expression profiles of the 2 distinct neoplastic components in this HCN-NOS tumor, correlating with the intratumoral heterogeneity identified histologically, and suggestive of tumor clonal evolution.

## Case Report

### Clinical Presentation and Surgical Findings

A previously healthy 3-year-old boy was found to have an 8.5 cm × 7 cm × 6 cm right liver mass by chest and abdomen CT scan. The patient underwent Tru-cut biopsy, diagnosed of fetal HB, and assigned a Pretreatment Extent of disease (PRETEXT) stage of II and an intermediate CHIC-HS risk score, due to the presence of multifocal disease. After 4 cycles of chemotherapy with cisplatin over 5 months, AFP levels dropped from 89,000 to 39,000 ng/mL. The patient underwent a right hepatectomy without complications. Post-surgery, CT scans showed no evidence of tumor, and AFP levels decreased to 19 ng/mL. The patient was doing well with no evidence of disease 1 year after completing chemotherapy.

### Histopathology and Immunohistochemistry

**Figure 1.**
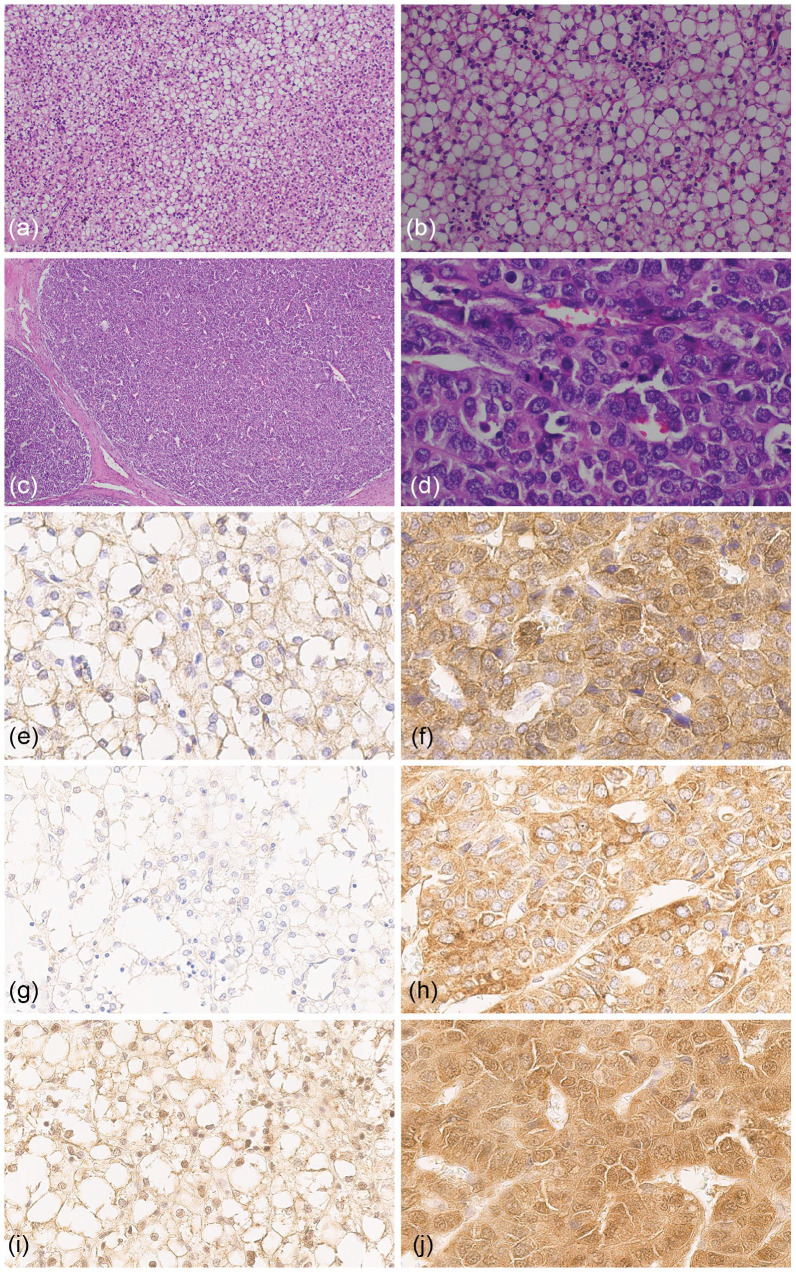
H&E examination of the HB-like component from biopsy specimen at 100× (a) and 400× (b), and distinct HCC-like nodule from resection specimen at 40× (c) and 400× (d). HB-like component immunohistochemistry (IHC) staining of β-catenin (e), AFP (g), LIN28B (i), compared to distinct HCC-like nodule IHC staining of β-catenin (f), AFP (h), and LIN28B (j).

Histological examination of biopsy demonstrated uniform hepatocellular elements arranged in trabeculae of 2–3 cells in thickness (Figure 1(a)). These hepatocytes resembled those of the developing fetal liver, with small round nuclei, indistinct nucleoli, and clear or finely granular cytoplasm containing a variable amount of glycogen and lipid (Figure 1(b)). Mitoses were absent. These findings were consistent with an epithelial hepatoblastoma with a fetal pattern. Post-chemotherapy specimen showed, in addition to the fetal HB component, HCC-like areas consisting of moderately differentiated atypical tumor cells with a high nuclear to cytoplasmic ratio, pleomorphic nuclei, decreased cytoplasmatic glycogen, and increased mitotic activity, arranged in trabecular and macrotrabecular growth patterns (Figure 1(c) and (d)). The adjacent non neoplastic liver was unremarkable and without underlying liver disease.

Immunohistochemistry demonstrated fetal HB areas (in both biopsy and resection specimens) showing mostly membranous and negative to only very focal nuclear expression of β-catenin, diffuse positivity for glutamine synthetase (GS), weak focal positivity for glypican 3 (GPC-3), IGF2, and LIN28B, and it was negative for AFP and HNF1A (Figure 1(e), (g), and (i)). HCC-like areas showed strong cytoplasmic and focal weak to moderate nuclear expression of β-catenin, focal for IGF2, diffuse positivity for GS, GPC-3, HNF1A, and LIN28B, and diffuse strong positivity for AFP (Figure 1(f), (h), (j)). The HB component was negative for SALL4 and SOX9 expression, while the HCC-like nodule was weakly positive for both.

### Cytogenetic and Molecular Findings

**Figure 2.**
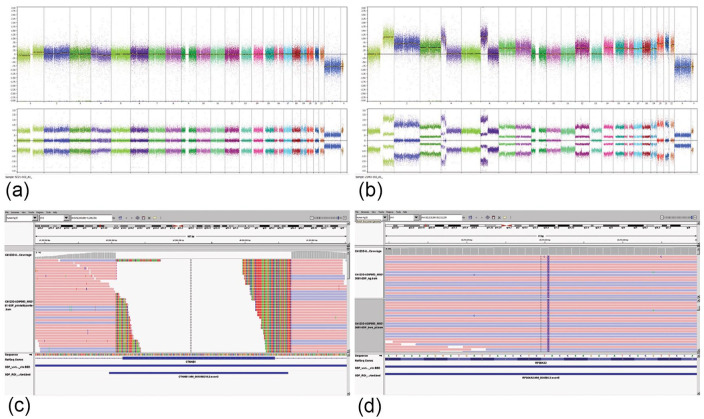
Copy number overview of HB-like component (a) and HCC-like nodule (b). Integrative Genomics Viewer (IGV) showed a c.14-10_241+25del variant in *CTNNB1* (catenin beta 1) at a VAF of 26% (c), and a c.364_368dup, p.Glu124TrpfsTer2 frameshift (p.E124fs) variant in *RPS6KA3* gene at a VAF of 67.5% (d).

Affymetrix Oncoscan copy number analysis of the HCC-like nodule revealed an unstable hyperdiploid genome with numerous chromosomal and segmental numerical abnormalities, including gains of chromosomes 1q, 2, 3, 4p, 6p, 7, 8, 12, 14, 15, 16, 17, 18, 19, 20, 21, and 22 (Supplemental Table S1). Copy neutral loss of heterozygosity (LOH) of 1p, 4q, 5, 6q, and 11p was also detected. In contrast, the nodule displaying fetal HB histology showed a stable genome with a very limited number of structural and numerical abnormalities including 1q gain, 1p loss, and segmental 4q loss (Figure 2(a) and (b)), all previously reported in HB.

Next-generation sequencing (NGS) using the Texas Children’s Hospital Pediatric Solid Tumor Cancer Mutation Panel^4^ was performed on DNA extracted from HB and HCC-like macro-dissected areas of the lesion. A *CTNNB1* c.14-10_241+25del (p.A5_A80del) variant at a variant allele fraction (VAF) of 26%, and a c.364_368dup, p.Glu124TrpfsTer2 frameshift (p.E124fs) variant of the *RPS6KA3* gene at a VAF of 67.5% were detected in the HCC-like component (Figure 2(c) and (d)). The HB nodule carried the same variants *CTNNB1* and *RPS6KA3* but at a much lower VAF (~5%), suggesting the evolution of a tumor subclone.

### mRNA Expression Profiling and IHC Protein Expression Levels

**Figure 3.**
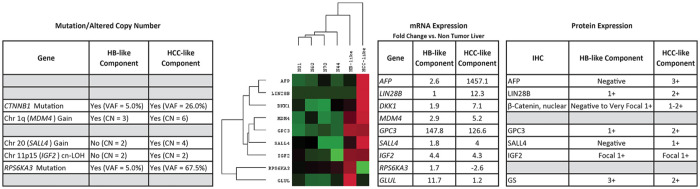
Relationship between gene mutations, gene copy number changes, mRNA expression levels, and protein expression of several prognostic biomarkers in HB-like and HCC-like components of an HCN-NOS tumor.

**Figure 4.**
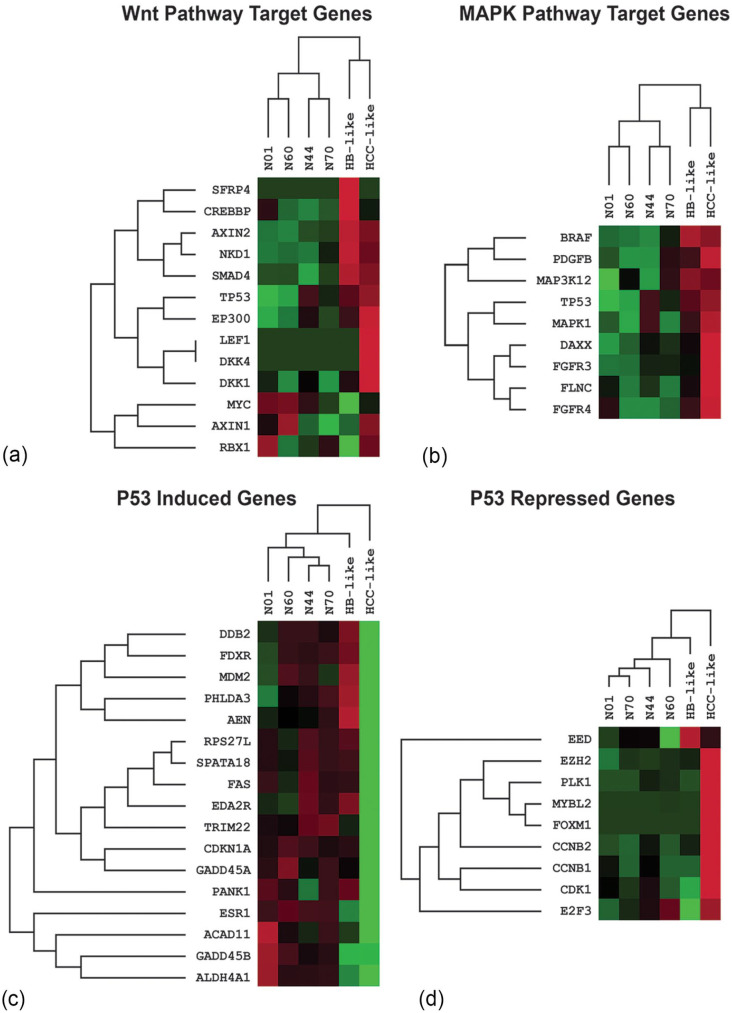
Gene expression heatmap of WNT pathway activation (a) MAPK pathway activation (b) and p53 pathway dysregulation (c and d) in HB-like and HCC-like components of a HCN-NOS tumor compared to non-tumor liver specimens.

mRNA profiling of the HB and HCC-like components was performed using a custom 635-gene NanoString expression panel designed to profile pediatric liver neoplasms (HB and HCC) cancer pathway activation, including WNT pathway genes, p53 activity, MAPK signaling, and oxidative stress, as well as housekeeping genes (Supplemental Table S2). Gene mutations and copy number changes detected in each of the histological component were correlated with expression changes in the corresponding genes and/or cancer pathway activation (Figures 3 and 4; Supplemental Table S3). The top 50 differentially expressed genes (compared to the adjacent non-tumoral liver) including over and under expressed genes for the HB and HCC-like components are listed in Supplemental Table S4. Both HB and HCC-like components demonstrated evidence of WNT pathway activation (Figure 4(a)) with overexpression of several WNT downstream target genes. These findings were consistent with the presence of the *CTNNB1* c.14-10_241+25del variant at VAF 5% and 26% in HB and HCC-like components, respectively, and IHC protein expression, which showed strong cytoplasmic and focal nuclear β-catenin expression in the HCC-like component.

Specific MAPK signaling in the HCC component was detected with overexpression of *DAXX*, *FGFR3*, and *FLNC* (approximately 2-fold, 8-fold, and 3-fold overexpression, respectively; Figure 4(b) and Supplemental Table S3). Interestingly, high-level copy gain (CN = 6) of both *DAXX* (6p21.3) and *FGFR3* (4p16.3) and low-level copy gain (CN = 3) of *FLNC* (7q32.1) were observed in the HCC component (Supplemental Table S1). Also, the expression of *RPS6KA3* (Xp22.12), which is downstream of MAPK1/ERK2 signaling and mediates inhibitory feedback, was reduced in the HCC-like component (approximately 2.6-fold; Figure 3).

The HCC-like component showed low expression levels of p53-induced genes and high expression levels of p53 repressed genes, indicating inactivation of the p53 pathway (Figure 4(c) and (d)). A summary of the top 50 differentially over and under expressed genes between HCC-like and HB-like components is presented in Supplemental Table S5.

Gene mutations, copy number alterations, and mRNA and protein expression levels are shown in Figure 3. The HCC-like component showed stronger expression of AFP, GPC3, LIN28B, SALL4, and lower GS expression by IHC, while IGF2 expression was similar in both HB and HCC-like components, consistent with mRNA expression levels. Elevated *MDM4* expression was greater in HCC-like component, correlating with 1q copy number gain. Reduced *RPS6KA3* mRNA expression in the HCC-like component is consistent with the *RPS6KA3* frameshift mutation detected at a high VAF in the HCC-like component.

## Discussion

Our results demonstrate biological heterogeneity and specific molecular abnormalities associated with the distinct histological patterns identified in 2 distinct components of this HCN-NOS. Increased mRNA and protein expression levels of biomarkers reported in high-risk HBs and in HCC, as well as a *RPS6KA3* mutation resulting in the induction MAPK pathway, were detected in the HCC-like component. Several WNT pathway targets were overexpressed in both HB and HCC-like components, consistent with the presence of a *CTNNB*1 deletion involving exon 3, and nuclear β-catenin overexpression detected by immunohistochemistry in both components.^6^ Activation of the WNT-signaling pathway occurs in the vast majority of HBs and a subset of HCCs through somatic mutations of *CTNNB1*, or rarely, other WNT-signaling genes such as *APC* and *AMER1.*^3^ Point mutations and in-frame deletions in the ubiquitination domain of *CTNNB1* generally involve exon 3,^6^ and result in the loss of phosphorylation sites within the E3 ubiquitin ligase recognition motif of the β-catenin protein.

Molecular analysis of the HCC-like component of the tumor, demonstrated a highly unstable hyperdiploid genome and evidence of altered cell cycle control, possibly associated with the *MDM4* copy gain (located in 1q32), as well as MAPK signaling pathway activation resulting from the *RPS6KA3* variant. *MDM4* has been reported to be amplified in a small subset of aggressive HBs, and over expressed in HCN-NOS and HCC.^4,7^ *RPS6KA3* gene (Xp22.12) encodes a serine/threonine-protein kinase, ribosomal S6 protein kinase 2 (RSK2), that acts downstream of MAPK1/ERK2 signaling to mediate feedback inhibition. RPS6KA3 also regulates mTOR pathway, phosphorylates, and activates p53 pathway.^8^ Inactivating mutations and copy loss of *RPS6KA3* have been reported in hepatocellular cancers, including HCN-NOS and HCC.^4,9,10^ HCCs commonly harbor inactivating *RSK2* mutations, resulting in attenuated a SOS1/2-dependent negative feedback loop, and leading to the activation of MAPK/ERK signaling.^11^ Therapeutically targetable activating mutations in MAPK/ERK signaling pathway genes, including *MAPK1* and *BRAF*, are clinically relevant, particularly for patients with relapsed or recurrent tumors who might be eligible for trials of molecularly targeted agents.^9^

We examined the expression of several markers by IHC and mRNA.^4,6^ High expression of LIN28B and SALL4 have been reported to be predictive biomarkers of poor prognosis in HB.^6^ LIN28B regulates stem-cell growth and metabolism. LIN28B expression levels have been highly correlated with increased expression of other oncofetal genes (such as AFP and GPC3),^6^ and possibly associated with a specific stage of developmental arrest. β-Catenin expression was uniformly high, consistent with the *CTNNB1* variant and previously documented driver role of WNT pathway activation in HB, HCN-NOS, and a subset of HCCs.^4,5^ Our mRNA expression results were consistent with DNA and protein expression changes detected, except for GPC3. *IGF2* mRNA was over-expressed in both HCC-like and HB-like components, however, 11p15 cn-LOH was only detected in the HCC-like component, suggesting that perhaps other mechanisms, such as recently reported epigenetic abnormalities in the HB component, may account for *IGF2* over-expression in the HB component.

Most HBs respond well to chemotherapy with favorable outcomes.^10^ However, the 3-year overall survival rate for high-risk HBs (including HCN-NOS) is below 50%, and guidelines for accurate classification, risk assessment, and subsequent therapeutic strategies, are still evolving. IHC is easily performed in routine pathology practice and is a useful screening tool, especially for highly expressed prognostic biomarkers. IHC, in combination with clinical, histological, and molecular markers, will improve risk-stratification of HCN-NOS patients, allowing for better therapy selection, and development of new therapeutic strategies that may improve the survival of these patients.

## Supplemental Material

sj-xlsx-1-pdp-10.1177_10935266231204788 – Supplemental material for Molecular Profiling of a Hepatocellular Neoplasm Not Otherwise Specified (HCN-NOS) Demonstrates Distinct Molecular Features in Hepatoblastoma and HCC-Like Components Supplemental material, sj-xlsx-1-pdp-10.1177_10935266231204788 for Molecular Profiling of a Hepatocellular Neoplasm Not Otherwise Specified (HCN-NOS) Demonstrates Distinct Molecular Features in Hepatoblastoma and HCC-Like Components by Yan Chen Wongworawat, Stephen F. Sarabia, Martin Urbicain, Paola Francalanci, Pavel Sumazin, Rita Alaggio and Dolores H. López-Terrada in Pediatric and Developmental Pathology

sj-xlsx-2-pdp-10.1177_10935266231204788 – Supplemental material for Molecular Profiling of a Hepatocellular Neoplasm Not Otherwise Specified (HCN-NOS) Demonstrates Distinct Molecular Features in Hepatoblastoma and HCC-Like Components Supplemental material, sj-xlsx-2-pdp-10.1177_10935266231204788 for Molecular Profiling of a Hepatocellular Neoplasm Not Otherwise Specified (HCN-NOS) Demonstrates Distinct Molecular Features in Hepatoblastoma and HCC-Like Components by Yan Chen Wongworawat, Stephen F. Sarabia, Martin Urbicain, Paola Francalanci, Pavel Sumazin, Rita Alaggio and Dolores H. López-Terrada in Pediatric and Developmental Pathology

sj-xlsx-3-pdp-10.1177_10935266231204788 – Supplemental material for Molecular Profiling of a Hepatocellular Neoplasm Not Otherwise Specified (HCN-NOS) Demonstrates Distinct Molecular Features in Hepatoblastoma and HCC-Like Components Supplemental material, sj-xlsx-3-pdp-10.1177_10935266231204788 for Molecular Profiling of a Hepatocellular Neoplasm Not Otherwise Specified (HCN-NOS) Demonstrates Distinct Molecular Features in Hepatoblastoma and HCC-Like Components by Yan Chen Wongworawat, Stephen F. Sarabia, Martin Urbicain, Paola Francalanci, Pavel Sumazin, Rita Alaggio and Dolores H. López-Terrada in Pediatric and Developmental Pathology

sj-xlsx-4-pdp-10.1177_10935266231204788 – Supplemental material for Molecular Profiling of a Hepatocellular Neoplasm Not Otherwise Specified (HCN-NOS) Demonstrates Distinct Molecular Features in Hepatoblastoma and HCC-Like Components Supplemental material, sj-xlsx-4-pdp-10.1177_10935266231204788 for Molecular Profiling of a Hepatocellular Neoplasm Not Otherwise Specified (HCN-NOS) Demonstrates Distinct Molecular Features in Hepatoblastoma and HCC-Like Components by Yan Chen Wongworawat, Stephen F. Sarabia, Martin Urbicain, Paola Francalanci, Pavel Sumazin, Rita Alaggio and Dolores H. López-Terrada in Pediatric and Developmental Pathology

sj-xlsx-5-pdp-10.1177_10935266231204788 – Supplemental material for Molecular Profiling of a Hepatocellular Neoplasm Not Otherwise Specified (HCN-NOS) Demonstrates Distinct Molecular Features in Hepatoblastoma and HCC-Like Components Supplemental material, sj-xlsx-5-pdp-10.1177_10935266231204788 for Molecular Profiling of a Hepatocellular Neoplasm Not Otherwise Specified (HCN-NOS) Demonstrates Distinct Molecular Features in Hepatoblastoma and HCC-Like Components by Yan Chen Wongworawat, Stephen F. Sarabia, Martin Urbicain, Paola Francalanci, Pavel Sumazin, Rita Alaggio and Dolores H. López-Terrada in Pediatric and Developmental Pathology
